# Comparison of Continuous Infusion of Ropivacaine and Fentanyl With Intermittent Bolus Doses of Ropivacaine and Fentanyl for Epidural Labor Analgesia: A Randomized Open-Label Study

**DOI:** 10.7759/cureus.28243

**Published:** 2022-08-21

**Authors:** Pallavee Priyadarshini, Reetu Verma, Premraj Singh, Shefali Gautam, Dinesh Singh, Monica Kohli, Shruti Kabi, Renu Singh

**Affiliations:** 1 Anaesthesiology, King George's Medical University, Lucknow, IND; 2 Obstetrics and Gynaecology, King George's Medical University, Lucknow, IND

**Keywords:** intermittent infusion, continuous infusion, ropivacaine, obstetric analgesia, local anaesthetics, labor pain

## Abstract

Background

The purpose of this study was to compare the efficacy of continuous epidural infusion with intermittent bolus doses for labour analgesia using ropivacaine 0.2% and opioids.

Methods

In this study, 70 primigravida patients were randomly divided into two groups of 35 each. Both groups received a loading dose of 10ml of 0.2% ropivacaine and 1μg/ml fentanyl in 5ml incremental doses while monitoring blood pressure and heart rate. Subsequently, Group I received a continuous epidural infusion of 0.2% ropivacaine with fentanyl at 10ml/hr, while Group II received 10 ml of 0.2% ropivacaine with fentanyl in bolus form every hour manually, with the first dose given after one hour of the initial loading dose. A rescue bolus dose of 5ml of 0.2% ropivacaine was given in both groups when they complained of breakthrough pain (VAS score >3). An additional 5ml bolus dose was given in both groups at the time of crowning. The blood pressure, heart rate, and severity of pain using the visual analogue scale (VAS) were assessed. Total drug volume utilized, the number of bolus doses, duration of the first and second stage of labour, rate of instrumental delivery and cesarean section, and neonatal Apgar scores were also recorded.

Results

The total volume of drugs consumed and the number of boluses required for breakthrough pain were both significantly lower in Group II. There was a similar decrease in hemodynamic parameters (systolic blood pressure, diastolic blood pressure, and mean arterial pressure) from baseline in both the groups with no significant difference between them. The onset of analgesia was significantly faster in Group I with both groups achieving optimum analgesia (VAS ≤ 3) within 20 minutes of the loading dose. Maternal motor blockade scores, the duration of the first and second stage of labour, the rate of instrumental delivery, cesarean section, and neonatal Apgar scores, did not show any statistically significant difference between the two groups.

Conclusion

Both techniques, i.e. continuous epidural infusion and intermittent epidural boluses are effective for providing labour analgesia. But consumption of drugs and episodes of breakthrough pain was higher in the continuous infusion group (Group I).

## Introduction

The use of epidural analgesia during childbirth is the gold standard as it provides adequate pain relief in both stages of labour. The use of a low concentration of local anaesthetic combined with lipid-soluble opioids provides optimal analgesia without delaying the progression of labour or affecting the mode of delivery and neonatal outcomes [1].

The mode of delivery is affected by the extent of motor block which in turn depends on the type, concentration, and method of administration of local anaesthetic along with the total consumption. The motor blockade affects the pelvic muscle tone, and the ability to "bear down" during the second stage of labour which directly affects the duration of labour, and instrumental deliveries [2].

The current standard labour epidural analgesic regimens consist of a local anaesthetic in combination with an opioid delivered via continuous epidural infusion (CEI) with or without patient-controlled epidural analgesia (PCEA) boluses. It has been observed that epidural bolus doses delivering the local anaesthetic in comparison to continuous infusion lead to a more extensive spread of the drug in the epidural space and hence, more sensory blockade [3,4]. Therefore, administration of the drug in regularly spaced intervals can help to reduce the dose of local anaesthetics. However, clinical data show varied results and do not support or disprove the hypothesis regarding the wider spread of local anaesthetic solution by intermittent bolus technique as the main mechanism of reduced analgesia consumption [4-6].

Thus, we have formulated a study to compare the efficacy of continuous epidural infusion with intermittent bolus doses for labour analgesia using a local anaesthetic (0.2% ropivacaine) plus opioid with the primary objective of comparing the total amount of drug consumption, and incidence of breakthrough pain (VAS>3) in both the groups. The secondary objectives of the study were to compare the duration of labour, number of cesarean sections, number of instrumental deliveries, and neonatal outcome.

## Materials and methods

**Figure 1:**
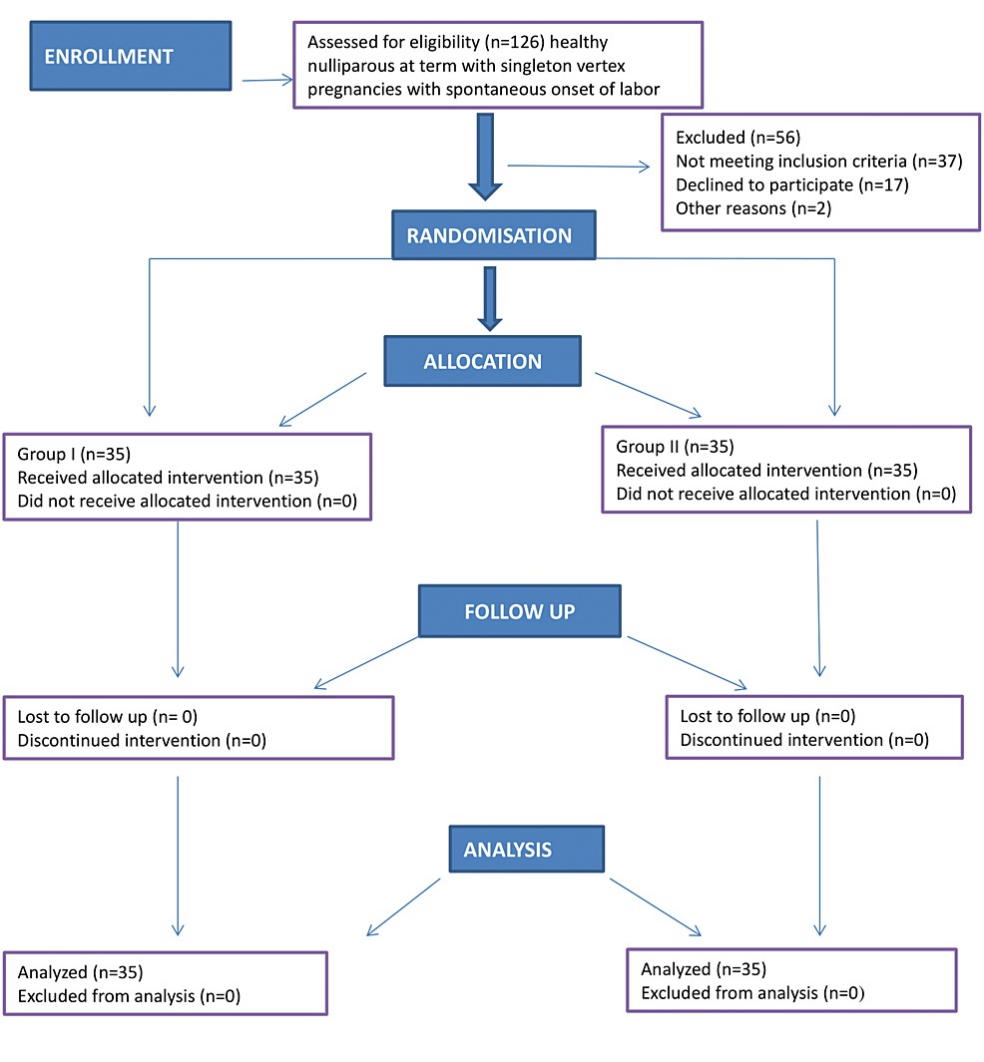
CONSORT flow diagram CONSORT: Consolidated Standards of Reporting Trials

After approval by the medical ethics committee (ECR/262/Inst/UP/2013/RR-19), this prospective randomized study was carried out on 70 primigravida hemodynamically stable patients (Figure 1). 

Written informed consent was taken from all patients. Inclusion criteria were ASA grade II parturient between 18-30 years of age, healthy term pregnancy who were in an active stage of labour with vertex presentation. Patients not giving consent, patients with systemic hypertension, pregnancy-induced hypertension, preeclampsia, eclampsia, diabetes mellitus, heart disease, previous lower segment cesarean section (LSCS) or any absolute indication for LSCS, allergy to study drugs, coagulopathy, infection at the site of epidural catheter insertion, evidence of spinal cord injury, and those with extremes of body weight or height were excluded.

After complete preoperative evaluation of the patients through history, examination, investigation, and obstetric consultation, all the patients were monitored for blood pressure, pulse rate, respiratory rate and fetal heart rate. The procedure was started in the first stage of labour, with regular uterine contractions and cervical dilation of less than 4cm. In every patient, intravenous access was achieved with a 20G peripheral cannula, and each patient was preloaded with 10ml/kg body weight Ringer’s lactate solution or Sterofundin 10-15 minutes before induction of epidural analgesia.

With proper aseptic precautions, under local anaesthesia (2% lignocaine), epidural space was identified with loss of resistance to air technique at L3-4 or L4-5 intervertebral space using an 18G Tuohy needle (Romsons Group, Delhi, India). Thereafter, a multi-hole epidural catheter was placed 4-5cm in the epidural space. To exclude intravenous or intrathecal catheter placement, a test dose of 3ml of 2% lignocaine with 1:200000 epinephrine (Cignoken^TM^ ADR 2%, Celon Labs, Hyderabad, India) was administered after negative aspiration for cerebrospinal fluid (CSF) and blood. If no toxicity reaction appeared, epidural analgesia was started. All patients were divided into two groups according to a computer-generated random number table. All the patients were given a 10ml bolus of 0.2% ropivacaine +1μg/ml fentanyl in 5ml incremental doses while monitoring blood pressure and heart rate, to begin with, after that Group I received a continuous epidural infusion of 0.2% ropivacaine with fentanyl at 10ml/hour and Group II received 10ml 0.2% ropivacaine with fentanyl in bolus form every hour manually. The first dose was given one hour after the initial loading dose. Patients in both groups were given a rescue bolus dose of 5ml of 0.2% ropivacaine if they complained of breakthrough pain (VAS score >3). At the time of crowning, an additional 5ml bolus dose was given in both groups. After giving the drugs, we checked the level of analgesia by pinprick using a 23G needle in a mid-clavicular line, every five minutes till the maximum level was achieved. VAS score was used to assess the severity of pain before the block and at 15, 30, 45, and 60 min and then at 30 min intervals. VAS was measured on a 0-10 scale where no pain was considered as 0 and the worst possible pain experienced was taken as 10. Motor block was assessed bilaterally using the modified Bromage scale (0 = no block, 1 = inability to raise the extended leg, 2 = inability to flex the knee and 3 = inability to flex ankle and foot). Motor block was assessed after the achievement of maximum sensory block and then at hourly intervals. Maternal heart rate, blood pressure, and oxygen saturation were measured non-invasively every five minutes for 15 minutes, then every 15 minutes for 45 minutes then every 30 minutes for 180 minutes or delivery of fetus whichever was early. Bradycardia (heart rate less than 50 per min) was treated with 0.5 mg atropine intravenous injection, which was repeated as needed. Any hypotension (systolic blood pressure (SBP) <90mmHg, or mean arterial pressure (MAP) <65 mmHg) was treated by intravenous infusion of normal saline and intravenous injection of mephentermine 6mg, which was repeated if needed.

The total dose of local anaesthetic required and the number of boluses needed for breakthrough pain (VAS>3) were noted. The onset of analgesia was defined as the time from epidural drug injection to the time of recording a VAS ≤ 3 during active uterine contraction. Time to reach maximum analgesia was defined as the time from epidural drug injection to the time of recording the lowest VAS of either 0, 1 or 2 during active uterine contraction. Duration of the first and second stage of labour, mode of delivery in the form of normal vaginal delivery, instrumental delivery or LSCS were observed. Neonatal Apgar scores at one minute and five minutes and side effects on the neonate were noted.

Statistics and sample size

Data were analysed using statistical software SPSS version 23 (IBM Corp., Armonk, NY). A chi-square test was used to compare categorical data. A Student’s t-test was used to compare continuous variables of two groups. P value <.05 was considered statistically significant. The sample size was calculated to be 32 in each group on the basis of variation in mean doses of two drugs to achieve similar VAS from a previous study [7]. So, in our study, we included 35 in each group with the possibility of a few dropouts. The following formula was used to calculate the sample size.

n=(z_a_ + z_b_)^2^ (s_1_^2^ + s_2_^2^)/d^2^

where s_1 _= 3.88, the SD of the first group dose to achieve similar VAS; s_2_ = 4.30, the SD of the second group dose to achieve similar VAS; d = the minimum mean difference considered to be clinically significant was assumed two; type I error α was taken 5% corresponding to 95% confidence level and type II error β was taken 10% for detecting results with 90% power of the study. 

## Results

The demographic profiles of the patients in both groups were comparable with regards to age, weight and body and height (P>0.05) (Table 1).

**Table 1:** Tabular presentation of mean age, weight, and height of study population Values are expressed as mean ± SD *= significant, #= non-significant

	Group-I (n=35)	Group-II (n=35)	t-value	p-value
Age (years)	25.4±3.6	24.74±4.53	0.6748	0.5021^#^
Height (cm)	152.6±3.1	150.29±17.71	0.7601	0.4498^#^
Weight (kg)	57.8±5.2	57.24±7.91	0.35	0.7274^#^

At baseline, all the haemodynamic parameters and VAS scores of the two groups were comparable. The mean cervical dilation at the onset of labour of patients of Group I and Group II were 2.53± 0.37cm and 3.01±0.22cm, respectively. The mean difference was statistically insignificant (p=0.1587). Sensory level up to T-10 was achieved in all patients. The mean total volumes of the drug required in Group I and Group II were 58.97 ± 3.88ml and 49.53±3.65ml, respectively. The mean difference was statistically significant (p<0.0001). Similarly, we observed more incidence of breakthrough pain in Group I than in Group II with Group I receiving a mean of 5.83 ± 1.07 boluses and Group II receiving a mean of 3.42 ± 0.45 boluses (Table 2). The difference was statistically significant (p<0.0001).

**Table 2:** Tabular presentation of the mean of the number of boluses and total dose received by patients in both groups Values are expressed as Mean ± S.D. * significant, # non-significant

	GROUP-I (N=35)	GROUP-II (N=35)	t-value	p-value
Total dose required (ml)	58.97±3.88	49.53±3.65	10.48	p<0.0001*
Bolus	5.83±1.07	3.42±0.45	12.28	p<0.0001*

After administration of the loading dose, there was a progressive decrease in VAS and VAS remained comparable in both the groups till delivery except at 10 min (p=0.0075) and 15 min (p=0.0062) (Figure 1).

**Figure 2:**
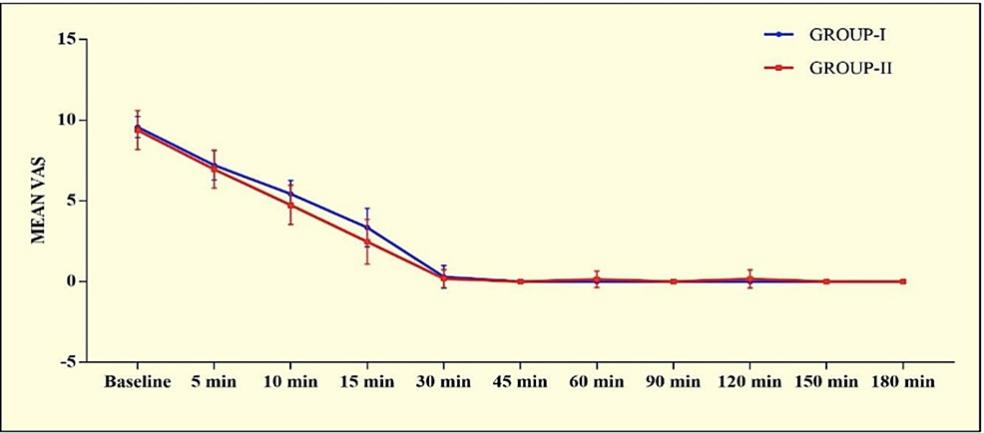
Graphical presentation of mean VAS score in both the groups

There also was a statistically significant difference in onset of analgesia with onset being significantly faster in Group I (P=0.0002). However, there was no significant difference in the duration of analgesia (Table 3).

**Table 3:** Tabular presentation of mean onset & duration of analgesia in both groups Values are expressed as mean ± SD. * significant, # non-significant

Onset and max analgesia	GROUP-I (n=35)	GROUP-II (n=35)	t-value	p-value
Mean onset of analgesia (min)	15.25±3.92	18.72±3.46	3.926	p=0.0002*
Duration of analgesia (min)	212.65 ± 26.62	204.87 ± 23.73	1.291	p=0.2012^#^

**Figure 3:**
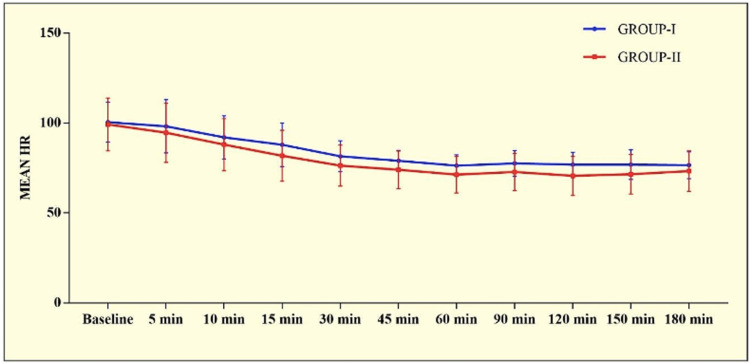
Graphical presentation of mean heart rate in the study population

**Figure 4:**
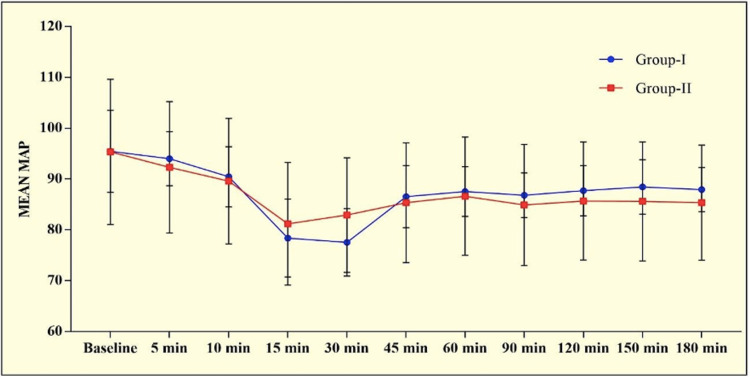
Graphical presentation of mean MAP (in mm of Hg) in the study population

In almost all the patients the modified Bromage score was 0 at all time intervals. Four patients in Group I and one in Group II showed motor involvement with a Bromage score of 1. On comparing heart rate, both groups had a gradually decreasing trend from the baseline till 1 hour after which became relatively stable till delivery. There was no statistically significant difference between the two groups (p>0.0005). Both groups showed a gradually decreasing trend in SBP, DBP and MAP that stabilized after 30 min of initiation of infusion and remained so till delivery. No significant inter-group difference was observed at any time interval (p>0.05) (Figures 3-4).

The incidence of LSCS was higher in Group I(14.29%) in comparison to Group II (2.86%) but it was statistically insignificant (p=0.20). There was no significant difference in the duration of the first and second stages of labour which remained normal in both groups. There were no significant fetal side effects in either group (Table 4).

**Table 4:** Tabular presentation of the duration of stages of labour, neonatal birth eight and Apgar scores in both groups Values are expressed as mean ± SD. * significant, # non-significant

	GROUP-I (N=35)	GROUP-II (N=35)	t-value	p-value
Duration of 1^st ^Stage (min)	240.57±31.92	234.59±29.68	0.8117	0.4198^#^
Duration of 2^nd ^Stage (min)	39.68±9.32	36.75±7.46	1.452	0.1511^#^
Birth weight (kg)	2.60±0.40	2.47±0.47	1.246	0.2170^#^
Apgar at 1 min	6.8±0.8	6.63±1.15	0.7179	0.4753^#^
Apgar at 5 min	8.5±0.6	8.38±1.18	0.5363	0.5935^#^

## Discussion

​​​​​In our study, we aimed to compare the efficacy of continuous epidural infusion with intermittent bolus doses using ropivacaine along with fentanyl as an adjuvant. We observed that patients in the intermittent bolus group had a lesser incidence of breakthrough pain and lower requirement of boluses in comparison to the continuous infusion group. The total cumulative dose of the drug was also less in the intermittent bolus group. Our results were consistent with other studies [8,9]. Gerhardt et al. also found that the number of boluses were significantly lower in the intermittent bolus group in comparison to the continuous infusion group [10]. However, they did not find any significant difference in the total volume of drugs consumed. Fidkowski et al. compared programmed intermittent bolus (PIEB) (5ml every hour and 10ml every hour) and continuous infusion (10ml/hour) of bupivacaine for labour analgesia and observed that patients with PIEB (10ml every hourly) had less incidence of breakthrough pain and required fewer drug boluses in comparison to PIEB (5ml every hour) and continuous infusion group [9]. Similar, to our study Sia et al. also observed that automated mandatory boluses result in reduced consumption of local anaesthetic in comparison to continuous infusion of drug [11]. The possible rationale behind higher consumption of the drug in the continuous group may be that during continuous infusion local anaesthetic exits only at the proximal hole of the catheter, so a very limited spread of drug occurs and hence a limited extent of block happens. But in the bolus injection technique, while giving drugs, higher pressure is generated which results in an even distribution of drugs through all holes of the multi-orifice epidural catheter and hence, a wider sensory block occurs [3,12].

However, in a study conducted by Ojo et al. using bupivacaine 0.125mg/ml and fentanyl 2mcg/ml, they found a higher ratio of patient-controlled epidural analgesia doses required per hour in the intermittent bolus group in comparison to the continuous infusion group but patients in PIEB group had less motor block [5].

In our study, we compared the efficacy of continuous epidural infusion with intermittent bolus doses using ropivacaine along with fentanyl as an adjuvant. We used 0.2% ropivacaine as studies comparing various concentrations of ropivacaine have shown 0.2% to have a faster onset and better quality of analgesia with fewer boluses needed [13,14]. The most used local anaesthetic for epidural analgesia is bupivacaine. However, ropivacaine is a long-acting amide that has lower lipophilicity than bupivacaine, less likely to penetrate big, myelinated motor fibres, resulting in a relatively mild motor block. Reduced lipophilicity relates to decreased risk of the central nervous system and cardiac toxicity [15]. With regards to motor involvement, four patients in Group I and one in Group II had a modified Bromage score of 1 with the remaining showing no motor involvement. This difference was statistically insignificant. In comparing VAS, we found that in both groups, the VAS decreased progressively and the onset of analgesia was achieved within 20 minutes of the loading dose. However, the mean onset was significantly faster in group I. We defined onset of analgesia as the time from epidural drug injection to the time of recording a VAS ≤ 3. VAS was comparable in both groups in our study at all times till delivery except at 10 and 15 minutes, however, this difference was not significant clinically. The rate of spontaneous vaginal deliveries, forceps, ventouse, and LSCS in the two groups were comparable with no statistically significant difference. Similar results were obtained by other studies [8,16,17]. But in a study by Capogna et al., the authors observed a significantly higher incidence of motor block and instrumental delivery in the continuous infusion group in comparison to the PIEB group [18].

There was no significant difference in the duration of the first and second stages of labour which remained normal in both groups. The Apgar scores of the neonates were similar and within the normal range in both groups. Overall, only one neonate in group I had NICU admission due to a low birth weight of 1.5 kg. On comparing hemodynamic parameters, HR, SBP, DBP and, MAP all showed a gradually decreasing trend after the loading dose that stabilized by 30 min and remained so till delivery in both the groups. None of the patients had bradycardia. We preloaded the patients to avoid the incidence of hypotension which was sufficient to avoid the incidence of transient sympathectomy-induced hypotension.

Limitations

The limitations of our research were the small sample size and that it was a single-centre study. Another limitation was the non-availability of a patient-controlled epidural analgesia infusion pump; hence bolus dose was given manually at the patients’ request. Also, both the interventionist as well as the observer were aware of the interventions that the patients had received.

## Conclusions

Both techniques, continuous epidural infusion and intermittent epidural boluses are effective for providing labour analgesia without any significant effect on hemodynamics. However, in comparison, the intermittent epidural bolus technique improved outcomes in many aspects: incidence of breakthrough pain was lower, fewer top-ups (boluses) were required and as well as fewer LSCS. We recommend further multi-centre studies to increase the reliability and generalizability of the study findings to further investigate possible differences in motor blocks and evaluate different parameters of programmed intermittent epidural boluses to optimize analgesia and outcomes.
